# Design and Rationale of the APELOT Trial: A Randomized, Open-Label, Multicenter, Phase IV Study to Evaluate the Antiplatelet Effect of Different Loading Dose of Ticagrelor in Patients With Non-ST Acute Coronary Syndrome Undergoing Percutaneous Coronary Intervention

**DOI:** 10.1097/MD.0000000000003756

**Published:** 2016-06-03

**Authors:** Hui-Liang Liu, Yu-Jie Wei, Zhi-Geng Jin, Jiao Zhang, Peng Ding, Sheng-Li Yang, Jian-Ping Luo, Dong-Xing Ma, Ying Liu, Wei Han

**Affiliations:** From the Division of Cardiovascular Diseases, General Hospital of Chinese People's Armed Police Forces, Haidian District, Beijing, China.

## Abstract

Ticagrelor is a direct acting on the P2Y12 receptor blocker, which provides faster and greater platelet inhibition than clopidogrel. However, several studies suggested that in ST-segment elevation myocardial infarction patients undergoing percutaneous coronary intervention (PCI), ticagrelor exhibits initial delay in the onset of antiplatelet action. Unlike ST-segment elevation myocardial infarction, in non-ST-segment elevation acute coronary syndrome (NSTE-ACS), management pathways are highly variable, and some patients may require surgery. Effect of higher loading dose (LD) of ticagrelor in patients with NSTE-ACS in providing faster and stronger inhibition of platelet aggregation is unknown and needs to be explored further.

The AntiPlatelet Effect of different Loading dOse of Ticagrelor trial is an interventional, randomized, open-label, multicenter, phase IV trial designed to evaluate whether a high LD (360 mg) of ticagrelor compared with the conventional LD (180 mg) will result in a higher inhibition of platelet aggregation without increasing bleeding events in NSTE-ACS participants undergoing PCI.

A total of 250 NSTE-ACS participants will be randomized to receive a ticagrelor LD (360 or 180 mg), followed by a maintenance dose of 90 mg twice a day (bid) starting 12 hours after the LD. The primary endpoint is platelet reactivity index measured by vasodilator-stimulated phosphoprotein phosphorylation 2 hours after the LD, and the secondary endpoints include occurrence of periprocedural myocardial infarction and bleeding events.

The AntiPlatelet Effect of different Loading dOse of Ticagrelor trial will provide important information on the risks and benefits of a high LD (360 mg) of ticagrelor in achieving a faster and stronger platelet inhibition compared with the conventional LD (180 mg) in NSTE-ACS patients undergoing PCI.

## INTRODUCTION

Rupture or erosion of an atherosclerotic plaque results in platelet activation, which is the key process in the formation of thrombus or embolus.^1^ Most cases of acute coronary syndrome are due to limited blood flow to the coronary muscles because of thrombotic occlusion of the artery. Non-ST elevation acute coronary syndrome (NSTE-ACS) usually occurs because of partial occlusion of a major coronary artery or complete occlusion of a minor coronary artery. The 1-year incidence of NSTE-ACS exceeds 1.5/1000 people, reflecting the substantial global healthcare burden from the disorder. Patients with NSTE-ACS are found to experience frequent recurrent ischemic events and a 2-fold higher death rate at 2 years despite optimal evidence-based therapy.^2–7^ Thrombotic complications after percutaneous coronary intervention (PCI) and recurrence of ischemic events in patients with ACS can be prevented by dual antiplatelet therapy with aspirin and clopidogrel. Early treatment with a P2Y12 receptor antagonist leads to a higher inhibition of platelet aggregation (IPA) in patients with unknown coronary artery anatomy before diagnostic coronary angiography and prevents recurrent atherothrombotic events in patients undergoing PCI. The 2007 European Society of Cardiology (ESC) level I-A recommends pretreatment with a loading dose (LD) of 300 mg of clopidogrel immediately after NSTE-ACS diagnosis, followed by a daily maintenance dose (MD) of 75 mg.^1^ However, in the present study, the LD of ticagrelor was not administered to patients. In the ONSET/OFFSET study, the onset and offset of antiplatelet effect of ticagrelor was statistically superior to high-dose clopidogrel (600 mg) in patients with stable coronary artery disease.^8^

Fast, uniform, and marked P2Y12 inhibition is recommended in patients with ST-segment elevation myocardial infarction (STEMI) undergoing PCI. The anti-ischemic effect of ticagrelor, with no excess bleeding, was superior to clopidogrel in the STEMI cohort in a subgroup analysis of the PLATelet inhibition and patient Outcomes trial. However, optimal IPA was rarely reached before 1 hour in a few studies. This effect may be because of specific conditions and a prethrombotic milieu that may modify the absorption, metabolism, and subsequent pharmacokinetics and pharmacodynamics of antiplatelet agents.^9,10^ More than one-half of the patients (n = 28) in a prospective, single-center, single-blind study had high platelet reactivity (HPR) at 1 hour of 180-mg (LD) ticagrelor treatment, demonstrating a delayed onset of antiplatelet action.^11^ Based on this observation, it is possible for patients with STEMI to theoretically achieve a faster platelet inhibition with a higher LD. In a prospective, 4-center, nonrandomized, controlled study, the authors hypothesized that doubling the standard 180 mg LD of ticagrelor might result in a higher drug concentration earlier, thereby providing a faster onset of antiplatelet activity. However, this hypothesis could not be confirmed, and doubling the LD of ticagrelor did not result in a faster onset of antiplatelet action than the standard dose.^12^

On the contrary, the degree of IPA achieved with a higher LD of ticagrelor in patients with NSTE-ACS remains to be explored. A recent systematic review and meta-analysis demonstrated a significant increase in major bleeding events and no significant reduction in mortality after pretreatment with thienopyridines in patients with NSTE-ACS.^2^

Significant global healthcare burden of NSTE-ACS and nonexistence of a standardized treatment regimen in patients with NSTE-ACS warrant more research in this domain. The AntiPlatelet Effect of different Loading dOse of Ticagrelor (APELOT) study intends to test the hypothesis that a high LD (360 mg) of ticagrelor will result in greater platelet inhibition than the conventional LD (180 mg) of ticagrelor in patients with NSTE-ACS undergoing PCI.

## METHODS

### Study Objectives

The APELOT trial (NCT01962428) will assess the hypothesis that a high LD (360 mg) of ticagrelor will result in greater platelet inhibition compared with the conventional LD of ticagrelor (180 mg) in NSTE-ACS patients undergoing PCI.

The primary endpoint is platelet reactivity index (PRI) measured by vasodilator-stimulated phosphoprotein phosphorylation (VASP-P) (2 h after the LD). The secondary endpoints include: the PRI measured by VASP-P at 0.5, 1, 8, and 24 hours after the LD; occurrence of periprocedural myocardial infarction (PMI); and occurrence of in-hospital and 1-month bleeding event (Bleeding Academic Research Consortium score types 1–5). The safety objective is to assess the safety and tolerability of different LDs of ticagrelor in Chinese patients with NSTE-ACS by evaluating adverse events. Additional exploratory objectives will compare the occurrence of in-hospital and 1-month death, myocardial infarction, stroke, and target vessel or target lesion revascularization with a double LD (360 mg) of ticagrelor.

### Study Design and Study Drug Administration

**FIGURE 1:**
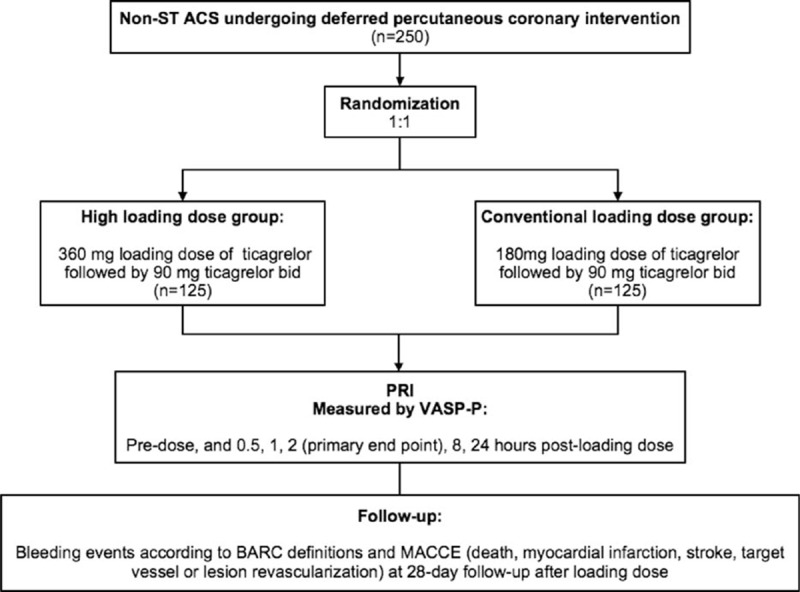
Study flow chart.

The APELOT trial is an interventional, randomized, open-label, multicenter, phase IV clinical trial that will recruit participants from 8 centers in Beijing, China. All centers would have highly trained experienced personnel. A total of 250 participants with NSTE-ACS who meet the entry criteria will be enrolled and randomized. The flow chart of the study design is shown in Figure 1.

After providing written informed consent, all participants will be randomized to receive a ticagrelor LD of 360 or 180 mg, and then a ticagrelor MD of 90 mg bid starting 12 hours after the LD. PCI will be performed within 2 to 72 hours after administration of the LD. All participants would receive acetylsalicylic acid 75 to 100 mg daily until they can tolerate. Glycoprotein IIb/IIIa receptor antagonists and low-molecular-weight heparin and other additional medication will be administered as directed by the treating cardiologist. All interventions will be performed via the radial approach with the standard technique within 72 hours after administration of the LD, and drug-eluting stents will be placed according to stenosis of the coronary artery. Blood samples of all participants will be obtained for the following assessments: PRI measured by VASP-P at predose and at 0.5, 1, 8, and 24 hours after the LD; and serum creatine kinase MB, troponin I, myoglobin, and C-reactive protein at predose, before PCI, and at 8 and 24 hours after PCI. ECG will be conducted at predose and at 8 and 24 hours after PCI. Incidences of PMI, in-hospital and 1-month bleeding events, death, myocardial infarction, stroke, and target vessel or target lesion revascularization will be documented. Participants will be followed up at the clinics or telephoned for 1 month.

#### Study Participants

The study population will consist of 250 participants with NSTE-ACS planning to undergo PCI. The diagnosis of patients with NSTE-ACS will be based on the 2011 ESC guidelines. The detailed inclusion and exclusion criteria are listed in Table 1.

**TABLE 1:**
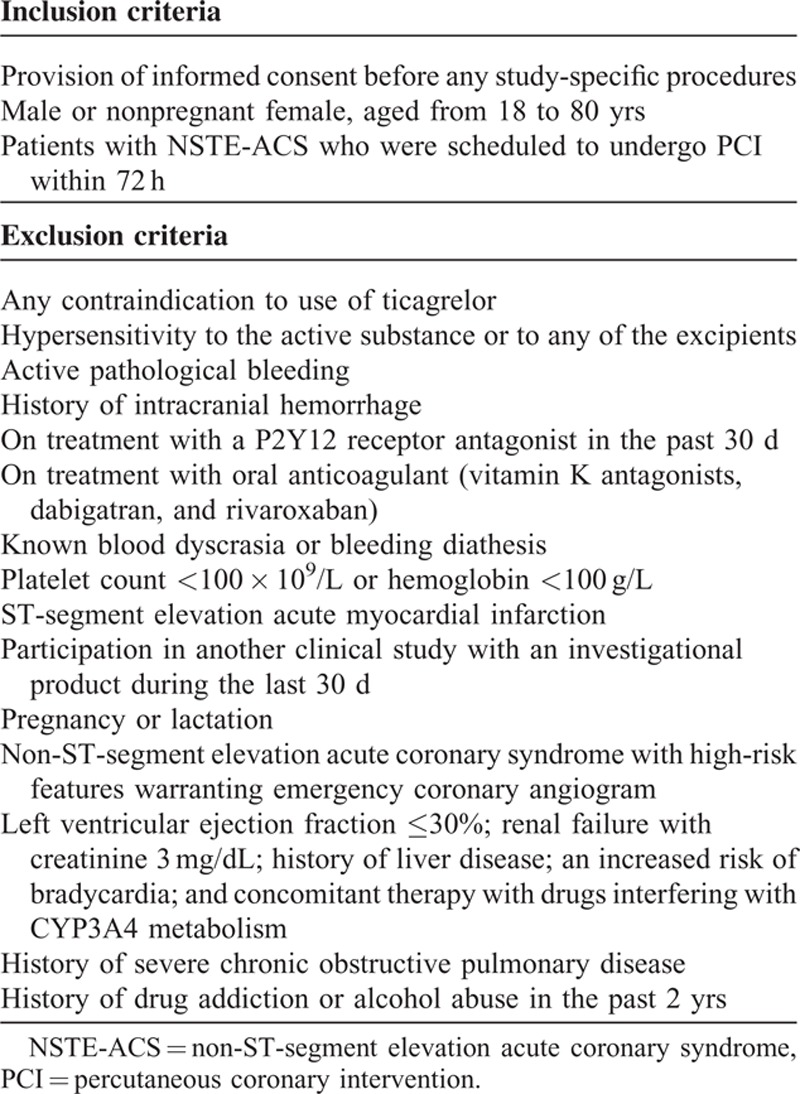
Patient Selection Criteria

The APELOT study will be performed in accordance with the ethical principles of the Declaration of Helsinki and will also be consistent with the International Conference on Harmonization and Good Clinical Practice. The study protocol, including the Informed Consent Form, is approved by an Independent Ethics Committee (IEC). The investigator will submit a written approval to the IEC before enrolling any patient into the study. The principal investigator(s) at each center will ensure that the patients were given adequate oral and written information about the purpose, and also possible risks and benefits of the study. Participants will also be notified that they are free to discontinue their participation at any time. The participants will be given the opportunity to ask any questions and time to consider the information provided. The participants’ signed and dated informed consent must be obtained before conducting any specific procedure during the study.

### Data analyses

#### Sample Size Justification

On the basis of onset/offset and respond studies, we expected that a double LD (360 mg) of ticagrelor will result in an absolute increase in IPA of 27% compared with the conventional LD (180 mg) of ticagrelor. With the assumption of a difference of 5.7 and a standard deviation (SD) of 16.344 PRI, and the covariate (baseline PRI) with an *R*^2^ value of 0.2, a total of 114 participants will be required in each arm to achieve a 90% power and a 2-sided alpha of 0.05. Considering a dropout rate of 10%, at least 250 participants would be required to complete the study to reach statistical significance.

#### Statistical and Analytical Plans

Continuous variables will be expressed as mean ± SD or median; and categorical variables will be expressed as frequencies and percentages. We performed statistical analyses using the SPSS version 22.0 (IBM Corp., Amrock, NY). We will use mixed-effects model to compare the pharmacodynamic assessments between the higher LD (360 mg) and conventional LD (180 mg) of ticagrelor. Goodness of fit of the model was assessed using regression and related techniques. Hazard ratio and 95% confidence interval estimates were provided using a Cox proportional-hazards model. Least-squares estimates of the mean difference are statistical significance, considered for *P* values <0.05.

### Implications

The APELOT trial will evaluate whether a high LD (360 mg) of ticagrelor will result in greater platelet inhibition compared with the conventional LD (180 mg) of ticagrelor in NSTE-ACS patients undergoing PCI. Despite optimal evidence-based treatment, NSTE-ACS participants were reported to have worse mid-term and long-term prognoses than STEMI participants with more frequent recurrent ischemic events and a 2-fold higher death rate at 2 years. Effective treatment strategy for such participants is yet to be established. If the APELOT trial confirms a higher LD of ticagrelor will have higher IPA and faster onset before PCI, it would help clinicians to choose the optimal antiplatelet therapy in patients with NSTE-ACS undergoing PCI. In addition, it would facilitate PCI procedures and improve clinical outcomes in patients with NSTE-ACS. The results of this study may have a direct impact on the future treatment for patients with NSTE-ACS, and may pave the way for future treatment recommendations.
